# Human Valacyclovir Hydrolase/Biphenyl Hydrolase-Like Protein Is a Highly Efficient Homocysteine Thiolactonase

**DOI:** 10.1371/journal.pone.0110054

**Published:** 2014-10-15

**Authors:** Judit Marsillach, Stephanie M. Suzuki, Rebecca J. Richter, Matthew G. McDonald, Peter M. Rademacher, Michael J. MacCoss, Edward J. Hsieh, Allan E. Rettie, Clement E. Furlong

**Affiliations:** 1 Department of Medicine (Division of Medical Genetics), University of Washington, Seattle, Washington, United States; 2 Department of Genome Sciences, University of Washington, Seattle, Washington, United States; 3 Department of Medicinal Chemistry, University of Washington, Seattle, Washington, United States; University Paris Diderot-Paris 7, France

## Abstract

Homocysteinylation of lysine residues by homocysteine thiolactone (HCTL), a reactive homocysteine metabolite, results in protein aggregation and malfunction, and is a well-known risk factor for cardiovascular, autoimmune and neurological diseases. Human plasma paraoxonase-1 (PON1) and bleomycin hydrolase (Blmh) have been reported as the physiological HCTL detoxifying enzymes. However, the catalytic efficiency of HCTL hydrolysis by Blmh is low and not saturated at 20 mM HCTL. The catalytic efficiency of PON1 for HCTL hydrolysis is 100-fold lower than that of Blmh. A homocysteine thiolactonase (HCTLase) was purified from human liver and identified by mass spectrometry (MS) as the previously described human biphenyl hydrolase-like protein (BPHL). To further characterize this newly described HCTLase activity, BPHL was expressed in *Escherichia coli* and purified. The sequence of the recombinant BPHL (rBPHL) and hydrolytic products of the substrates HCTL and valacyclovir were verified by MS. We found that the catalytic efficiency (k_cat_/K_m_) of rBPHL for HCTL hydrolysis was 7.7 × 10^4^ M^−1^s^−1^, orders of magnitude higher than that of PON1 or Blmh, indicating a more significant physiological role for BPHL in detoxifying HCTL.

## Introduction

Elevated homocysteine (Hcy) levels have been associated with cardiovascular disease [1], [2], diabetic retinopathy [3], birth defects, osteoporosis, renal failure [4] and Alzheimer' disease (AD) [5], [6]. Severe hyperhomocysteinemia is rare, but mild hyperhomocysteinemia is common in the general population. Hcy, a homologue of the amino acid cysteine, is generated from methionine following demethylation and can be converted back to methionine or to cysteine. In humans, Hcy serves as a precursor for maintaining the levels of methionine and cysteine. If methionine is needed, Hcy is methylated through two enzymes from the methionine and folate cycles with the aid of the co-factor vitamin B12. On the other hand, if there is an excess of methionine, Hcy is converted to cysteine via transsulfuration, which requires vitamin B6. Although Hcy can be synthesized in all human organs, most of it is detoxified in the liver and kidneys, organs that possess all the enzymes involved in the methionine metabolism [7]. Hcy is also converted to homocysteine thiolactone (HCTL) through error-editing reactions catalyzed by methionyl tRNA-synthetase. Homocysteine thiolactonase (HCTLase) activities hydrolyze HCTL to Hcy. Both Hcy and HCTL have been linked to many disease processes, however, HCTL appears to be the more toxic [4], [8]–[10] and has been shown to be neurotoxic in mice [9], [10].

HCTL is a reactive metabolite that modifies proteins by forming an isopeptide bond with the ε-amino groups of lysine residues [11]. Homocysteinylated proteins have the potential to misfold and aggregate which can activate the adaptive immune system and increase clot resistance to lysis, promoting atherosclerotic lesion formation. Homocysteinylated proteins may also lose biological activity [4], [11]. Therefore, *in vivo* homocysteinylation of proteins has been suggested as the mechanism by which elevated Hcy levels contribute to cardiovascular, neurological and autoimmune diseases [11]. HCTL adducts have been described on several plasma proteins, such as albumin, hemoglobin γ-globulin, high-density lipoproteins (HDLs), low-density lipoproteins (LDLs) and fibrinogen [12], [13]. Deficits in the enzymes metabolizing Hcy and HCTL can result in increased physiological concentrations of these metabolites and associated detrimental effects. Methylenetetrahydrofolate reductase (MTHFR), methionine synthase and cystathionine beta-synthase (CBS) are responsible for recycling Hcy back to amino acids. MTHFR is required to form 5-methyl tetrahydrofolate, a metabolite necessary for the conversion of Hcy to methionine. CBS is required to convert Hcy to cysteine. Deficiencies in any of these enzymes can result in increased levels of Hcy and HCTL (reviewed in [1], [14].

Human plasma paraoxonase-1 (PON1), a polymorphic high-density lipoprotein (HDL)-associated enzyme, synthesized primarily in liver and secreted to associate with HDL, hydrolyzes a number of lactones, including HCTL [15]–[17]. We have shown previously [18], [19] that the physiological significance of PON1 in detoxification of organophosphorus (OP) insecticides is highly dependent on the catalytic efficiency of hydrolysis of a given OP substrate. For example, while the catalytic efficiency of PON1_R192_ is ≈9-times greater than that of PON1_Q192_ for hydrolysis of paraoxon, it provides little if any protection against paraoxon exposure whereas PON1 protects against exposures to chlorpyrifos oxon and diazoxon [18]. The reported low substrate affinity and very low specific activity for hydrolysis of HCTL by PON1 [15], [16] have raised doubts about the physiological relevance of this activity [15], [20]. However, studies with *PON1^−/−^* mice provided some evidence that HCTL as opposed to Hcy is neurotoxic *in vivo* and that PON1 could protect against the neurotoxicity of HCTL [9]. For HCTLase measurement, two substrates are commonly used, HCTL and **γ**-thiobutyrolactone (GTBL), the latter with the rationale that HCTL is unstable and/or because of albumin interference with the Ellman assay [21]. Despite the very low catalytic efficiency of PON1 for hydrolyzing HCTL [8], [15], [20], PON1 has been referred to as plasma HCTLase [22].

Another HCTLase has been purified from human placenta and identified as bleomycin hydrolase (Blmh) [8]. This enzyme, first studied for its ability to hydrolyze the anti-cancer drug bleomycin, has been detected in testis, skeletal muscle, pancreas, spleen, and at lower levels in thymus, prostate, ovary, small intestine, heart, brain, placenta and lung with very low levels of expression observed in liver, kidney, colon and peripheral blood leukocytes [23]. The catalytic efficiency of human Blmh for hydrolysis of HCTL was reported to be ≈100-fold greater than that of PON1, even though it was not saturated at 20 mM HCTL [8]. HCTL administered to yeast or mice was shown to be toxic, with yeast Blmh (referred to as BLH1) preventing HCTL toxicity in yeast [8] and mouse Blmh protecting mice from HCTL toxicity [10]. Yeast *BLH1* (also known as *LAP3*) mutants and *Blmh* ^−/−^ mice exhibited increased sensitivity to HCTL and *Blmh* ^−/−^ mice demonstrated deficits in spatial memory [24] as well as increased susceptibility to HCTL toxicity [10]. Blmh has also been associated with AD, based in part on its ability to process β-amyloid peptides [24], [25]. HCTLase activity attributed to Blmh differed significantly between brains of AD patients and controls [5].

Biphenyl hydrolase-like protein (BPHL), also called valacyclovir (VC) hydrolase or valacyclovirase, is a 32,543 Da protein highly expressed in human liver and kidney and at lower levels in the intestine, heart and skeletal muscle [26], [27]. BPHL hydrolyzes and activates the antiviral prodrug esters VC and valganciclovir, which are used in the management of herpes simplex, herpes zoster (shingles) and herpes B [28]. First cloned from breast carcinoma cells, BPHL, a member of the alpha/beta hydrolase fold family, is a serine hydrolase distantly related to other members of the serine hydrolase family [26], [27]. BPHL has very low activity against standard esterase substrates such as *p*-nitrophenyl acetate/butyrate but higher activity for hydrolysis of a number of amino ester prodrugs [29]. Kim and coworkers have expressed and purified recombinant BPHL (rBPHL) from *Escherichia coli* (*E.coli*) [28]. Lai et al. reported a crystal structure of BPHL, which revealed a serine hydrolase consensus sequence of GSXSG and a catalytic triad at S122-D227-H255 [30]. A physiological function of BPHL has not yet been described [30], [31].

In this study, we report the purification of a human hepatic enzyme that demonstrates high HCTLase activity and was identified as BPHL by mass spectrometry (MS). When expressed in *E. coli*, the purified rBPHL exhibited high HCTLase activity. Both native and recombinant purified BPHLs have much higher catalytic efficiencies for HCTL hydrolysis than described for purified plasma PON1 or Blmh, making BPHL a much more likely candidate for protecting against HCTL toxicity under physiological conditions.

## Materials and Methods

### Enzyme assays

HCTLase activity was measured using a modification of the Ellman cholinesterase method [32]. A fresh solution of 5,5'-dithiobis-(2-nitrobenzoic acid) (DTNB) (10.3 mM) was prepared in 100 mM NaPO_4_, pH 7.0, and was added at a final concentration of 0.32 mM to a solution of 3 mM L-HCTL, in Dulbecco's PBS, with the volume dependent on the number of assays to be carried out. The substrate/DTNB solution (200 µL) was added immediately to enzyme samples (5–20 µL) in 96-well microplates. The assay was monitored at 405 nm for 4 min at 25°C for column fractions or at 37°C for kinetic analyses using a SPECTRAmax PLUS Microplate Spectrophotometer (Molecular Devices, Sunnyvale, CA). Inhibitors were added to the assays at levels indicated in the figures. GTBLase activity was measured by preparing a 28 mM solution of GTBL in Dulbecco's PBS. Just prior to use, a fresh solution of DTNB (10.3 mM) was prepared and added as above. The substrate and DTNB solution (200 µL) were added immediately to enzyme samples (5–20 µL) in 96-well microplates. The assay was monitored at 405 nm for 4 min at 37°C. Activities are expressed in Units/mL or Units/mg protein, based on the molar extinction coefficient of 14.15 mmol/L^−1^cm^−1^ for the hydrolysis of DTNB to 2-nitro-5-thiobenzoic acid (NTB) [33]. Only the initial linear rates of hydrolysis were used for calculations. Clopidogrel thiolactone, prasugrel thiolactone and the exo-thiol metabolites were a generous gift of Eli Lilly. Unless otherwise noted, all other reagents were obtained from Sigma-Aldrich (St. Louis, MO).

### Albumin and DTNB

**Figure 1:**
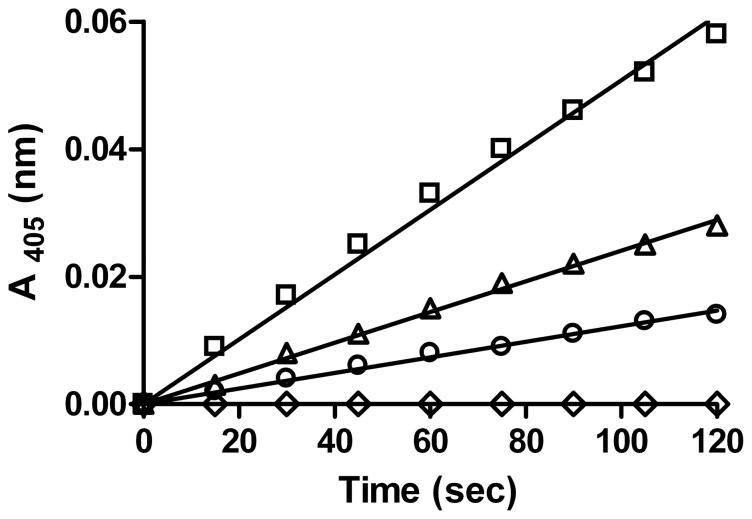
Reaction of human serum albumin (HSA) with Ellman's reagent (DTNB). Recombinant HSA (500 µg from *Pichia pastoris*) □―□; human plasma (10 µL) Δ―Δ; antibody purified HSA (200 µg) ○―○; no protein control ⋄―⋄.

To examine the basis of reported albumin interference of the Ellman assay [21], we monitored the rate of appearance of NTB in the absence of added substrate. Both purified and recombinant serum albumin exhibited a rate of appearance of NTB, characteristic of the enzymatic hydrolysis of thiolactones and thio-esters (Figure 1). Thus, it is important to be cognizant of this reaction when measuring rates of formation of free sulfhydryl groups in samples containing albumin or other proteins with free sulfhydryl groups. The assay of albumin with DTNB alone has apparent activity similar to that observed with PON1 in the presence of HCTL, so controls must be performed when using DTNB and serum or plasma samples to correct for the “albumin interference”. A concentration of 10 mM EDTA did not affect the rates of the DTNB/serum assay.

### Purification of human liver HCTLase

Liver tissue was obtained from the Human Liver Bank maintained within the School of Pharmacy at the University of Washington [34]. De-identified samples were obtained from deceased, anonymous individuals with approval of the Institutional Review Board (IRB) at the University of Washington. A 33 g human liver sample (pooled from five individual donors) was homogenized in 76 mL of 20 mM Tris-HCl (pH 8.0) buffer on ice using a Polytron tissue homogenizer. The sample was centrifuged at 17,000 × g in a Sorvall RC5B centrifuge for 40 min at 4°C. The supernatant was centrifuged at 424,000 × g in a tabletop ultracentrifuge (TL-100, Beckman Coulter, Brea, CA) for 30 minutes at 4°C, using a fixed-angle rotor (TLA 100.3, Beckman Coulter).

The soluble supernatant fraction (58 mL) was loaded onto a 25 mL DEAE (diethylaminoethyl) Sepharose Fast Flow (GE Healthcare, Piscataway, NJ) column (2.8 × 5.5 cm) equilibrated with 20 mM Tris-HCl, pH 8.0. The HCTLase activity did not bind to the column. The flow-through fractions with HCTLase activity were adjusted to pH 7.0 with 0.1N HCl, then loaded onto a 20 mL column of CM (carboxymethyl) Sepharose Fast Flow (1.8 × 8 cm) (GE Healthcare) that had been equilibrated with 20 mM Tris-HCl, pH 7.0 (CM buffer). The column was washed with 6 resin bed volumes (rbv's) of CM buffer, then eluted with 20 rbv's of a 0–600 mM NaCl linear gradient in CM buffer. Fractions with HCTLase activity were pooled and loaded onto a 2 mL ceramic hydroxyapatite (HA) column (1 × 4 cm, HA type II 40 µm, BioRad, Hercules, CA) that was equilibrated with 5 mM KPO_4_, pH 6.9. The HA column was washed with equilibration buffer until the A_280_ reached baseline level, then eluted with a 25 rbv gradient from 5–200 mM KPO_4_, pH 6.9. HCTLase-containing fractions were pooled and concentrated to 0.5 mL with a 10,000 molecular weight cut off (MWCO) Amicon Ultra centrifugal filter (Millipore, Billerica, MA) at 1,500 × g, loaded onto a 25 mL Superdex 200 (GE Healthcare) (1.2 × 32 cm) column equilibrated with 20 mM Tris-HCl (pH 7.0), 100 mM NaCl (equilibration buffer) and eluted with the same buffer. HCTLase-containing fractions were pooled, diluted 1∶2 with 20 mM Tris-HCl, pH 7.0 and loaded onto a 0.5 mL CM Sepharose column (0.5 × 3 cm) equilibrated with CM buffer. The column was eluted with a gradient from 0–600 mM NaCl in 20 rbv's of CM buffer. The fractions containing HCTLase activity were pooled and concentrated to 0.2 mL on a 10,000 MWCO Amicon Ultra concentrator at 1,500 × g, then loaded onto a 5 mL Superdex 200 column (0.8 × 22 cm) in 20 mM Tris-HCl, pH 8.0. Fractions containing HCTLase activity were pooled and stored at 4°C. Protein concentrations were measured with the BCA Protein Assay (Thermo Fisher Scientific Inc., Waltham, MA).

### In-gel activity and Coomassie Blue staining

Human liver purified BPHL and rBPHL (described below) were in-gel activity stained using an adapted protocol from Korenovsky et al. [35]. Both enzymes were first buffer exchanged into deionized water using Zeba spin desalting columns (Pierce Chemical, Rockford, IL) and then surface loaded on a 4.5 × 8 cm native IsoGel agarose isoelectric focusing (IEF) gel with a pH range of 6–10.5 (Lonza, Walkersville, MA). The anode solution used was 10 mM acetic acid (Thermo Fisher Scientific), and Cathode Fluid 10 was used as the cathode solution (SERVA Electrophoresis GmbH, Heidelberg, Germany). The IEF gel was run at 4°C with the voltage increased up to 60 V/cm over a period of 50 min (250 V maximum). Lysed human red blood cells provided a visible hemoglobin marker to ensure that the focusing of the proteins was completed. Following electrophoresis, half of the gel was used for HCTLase activity stain and the other half for Coomassie Blue stain. For the activity stain, the gel was washed with deionized water for 5 min, then equilibrated with reaction buffer [0.1 M sodium acetate, 20 mM glycine (Sigma-Aldrich), 4 mM cupric sulfate, 24% (w/v) sodium sulfate and 3 mM magnesium chloride (remaining reagents were from J.T. Baker Chemicals-Avantor Performance Materials, Center Valley, PA)] without substrate for 2 min. Then, the gel was incubated with 3.8 mM HCTL (Sigma-Aldrich) in reaction buffer with shaking for 1 h at room temperature. Following incubation with substrate, the gel was washed in deionized water for 5 min followed by a 3 M ammonium sulfate (Sigma-Aldrich) wash for 5 min. The gel was developed with 0.16% w/v dithio-oxamide in deionized water (Fluka, Sigma-Aldrich) until appearance of brown activity bands of copper- thio-oxamide was observed. For Coomassie Blue staining, the gel was fixed for 15 min in a solution of 36% (v/v) methanol, 6% (w/v) trichloroacetic acid and 3.6% (w/v) sulfosalicylic acid (all from Sigma-Aldrich). After rinsing with deionized water, the gel was stained with Coomassie Blue stain solution [0.1%(w/v) Coomassie brilliant blue (Amresco, Solon, OH), 25% (v/v) ethanol and 9% (v/v) acetic acid (both from Thermo Fisher Scientific)] overnight at room temperature and de-stained in 25% (v/v) ethanol with 9% (v/v) acetic acid for 3 h.

### Inhibition assays

Human liver homogenates were incubated with specific inhibitors for BPHL, Blmh or PON1. Briefly, 1 g of frozen human liver was thawed and homogenized on ice using a Tissue-Tearor hand-held homogenizer (Cole-Parmer, Vernon Hills, IL) in 1.5 mL of 20 mM Tris pH 8.0 supplemented with 1 mM CaCl_2_ (to preserve PON1 activity). Liver homogenates were centrifuged as described above. The obtained supernatants were incubated for 10 min with BPHL inhibitors valacyclovir (20 mM) or L-proline benzyl ester (10 mM); Blmh inhibitors E-64 (50 µM) or iodoacetamide (2 mM); and PON1 inhibitors EDTA (10 mM) or 2-hydroxyquinoline (200 µM). Then, HCTL hydrolysis was monitored as described above but using 10 mM HCTL as substrate.

### Cloning of human liver BPHL

A clone of human liver BPHL (BC106901) was obtained from Source Bioscience (Nottingham, UK). The 770 bp BPHL gene was amplified with primers containing NdeI restriction sites [(forward) CAG CAG CCA TGG GCA TGC CCA GGA ATC TGC TT] and NcoI [CAG CAG CAT ATG TCA TTG TAG GAA GTC TTC TGC (reverse)]. The resulting PCR product was cloned using the Invitrogen/Life Sciences TOPO TA Cloning Dual Promoter Kit, following the manufacturer's protocol. A pET15 expression vector (EMD Millipore, Billerica, MA) and the TOPO-BPHL clone were digested with NdeI and NcoI, then run on a 1.2% double tier FlashGel DNA Cassette (Lonza). The digested pET15 and the BPHL DNA fragment were eluted according to the manufacturer's protocol. Following DNA elution, they were ligated with T4 Ligase (NEB, Ipswich, MA), then transformed into XL10-Gold competent subcloning cells (Agilent, Santa Clara, CA). The pET15-BPHL insert was sequenced to verify the fidelity of the sub-cloning protocol. The sequence-verified construct was transformed into Rosetta-Gami 2(DE3) expression cells (EMD Millipore).

### Expression of rBPHL

Two 25°C-overnight cultures (600 mL and 800 mL) of Rosetta-Gami 2(DE3) cells transformed with the pET15-BPHL were inoculated respectively into two bubble jars containing 6 L and 8 L of LB media at 25°C with carbenicillin at 50 µg/mL. Cells were induced at an A_600_ of 0.6 with 1 mM IPTG and grown for an additional 12 h. Cells were harvested by centrifugation at 4,800 × g for 20 min at 4°C. Aliquots (0.1 g) of induced and uninduced cells were solubilized with BPER II Protein Extraction Reagent (Pierce Chemical) for analysis of HCTLase activity. The remaining induced cells (19 g) were frozen at -20°C.

### Purification of rBPHL

Frozen, induced *E*. *coli* cells (9.6 g) were thawed on ice, then lysed and extracted with 2 volumes (w/v) of B-PER II containing 20 Units/mL of Benzonase (EMD Millipore) and Protease Inhibitor Cocktail Set III, EDTA-Free (EMD Millipore) diluted 1∶200. The lysed, resuspended cells were gently mixed and incubated at room temperature for 30 min. Insoluble particulate was removed by centrifugation at 12,000 × g for 20 min at 4°C. The soluble supernatant fraction was loaded onto a 10 mL DEAE Sepharose Fast Flow column (1.5 × 6 cm) equilibrated with 20 mM Tris-HCl (pH 8.0) buffer. The HCTLase activity did not bind to the DEAE resin. The pool of the flow-through fractions containing HCTLase activity was adjusted to pH 6.9 with 0.1 N HCl and loaded onto a 10 mL ceramic HA column (1.2 × 8 cm) then washed with 5 mM KPO_4_ (pH 6.9) buffer, and eluted with a 20 rbv 5–225 mM KPO_4_ gradient. The active fractions were pooled and concentrated to 1.0 mL with a 10,000 MWCO Amicon Ultra centrifugal filter at 1,500 × g, then loaded onto a 75 mL Superdex 200 (1.8 × 45 cm) column equilibrated with 20 mM Tris-HCl (pH 8.0), 100 mM NaCl and eluted with the same buffer. Fractions containing HCTLase activity were pooled for gel analysis.

### Mass spectrometric analysis of the purified proteins

The purified liver BPHL and rBPHL were analyzed by high-resolution MS. Five µg of the purified hepatic protein were loaded to a denaturing NuPAGE 4–12% Bis-Tris gel (Life Technologies, Grand Island, NY). After staining the gel with Imperial Protein Stain Solution (Thermo Fisher Scientific), three bands were excised for protein sequencing by MS. The excised bands were reduced in-gel with 10 mM dithiothreitol (DTT) and alkylated with 55 mM iodoacetamide (IAA). The carbamidomethylation step was followed by in-gel digestion with 2 µg of porcine trypsin (Promega, Madison, WI), with shaking at 37°C overnight. The generated tryptic peptides were extracted with 5% formic acid (v/v): acetonitrile (1∶2), evaporated to near dryness (CentriVap vacuum concentrator, Labconco SpeedVac, Kansas City, MO) and reconstituted in 20 µL of 2% acetonitrile, 0.1% formic acid.

Five µg of the purified rBPHL were digested in solution. The surfactant RapiGest SF (Waters Corporation, Milford, MA) was added to a final concentration of 0.1% and the sample was reduced with 5 mM DTT and alkylated with 15 mM IAA. Then, the samples were digested with 1 µg of porcine trypsin, shaking at 37°C for 2 h. After the digestion, hydrochloric acid was added to a final concentration of 100 mM to hydrolyze the surfactant. The white precipitate formed was separated by centrifugation.

Both extracted peptides and peptides obtained by in-solution digestion were separated by nanoflow chromatography in a Waters nanoACQUITY UltraPerformance liquid chromatography (UPLC) system (Waters Corporation). A 20 cm, 75 µm fused I.D. silica capillary column was used, with a 5 µm I.D. tip pulled using a P-2000 CO_2_ laser puller (Sutter Instrument Company, Novato, CA). The column was packed in-house to the stated length with Jupiter 4 µm Proteo 90Å C12 reversed-phase resin (Phenomenex, Torrance, CA). The peptides were eluted using a 50 min gradient of 2–32% acetonitrile gradient in 0.1% formic acid, at 250 nL/min, and analyzed by an LTQ-Orbitrap (Thermo Fisher Scientific). The SEQUEST algorithm [36] was used for database searches of the tandem MS output, with a 57.021464 Da static carbamidomethyl modification added to cysteine residues.

### Mass spectrometric analysis of the products of HCTL hydrolysis

HCTLase (1 or 10 ng; for VC or HCTL, respectively) was pre-incubated in PBS buffer at 37°C and shaken at 70 rpm in a water bath for 2 min prior to addition of substrate (VC or HCTL at 100 µM final concentrations) in a 200 µL reaction volume. Reactions were quenched at time points of t  =  0 and t  =  5 min with the addition of 20 µL of a 15% aqueous ZnSO_4_ solution on ice, then centrifuged to remove protein and buffer salts. Supernatants were analyzed by LC-MS/MS.

Reaction products were analyzed with a Micromass Quattro Micro API, Tandem Quadrupole Mass Spectrometer (Waters Corporation) equipped with a Shimadzu HPLC system, consisting of two LC-10AD pumps, an SCL-10Avp controller and an SIL-10ADvp autosampler (Shimadzu Scientific Instruments, Inc., Columbia, MD). The MS was run in positive electrospray ionization mode at a source temperature of 120°C and a desolvation temperature of 330°C. The cone voltage was set at 20 volts and the collision energy was set to 20 eV. The following mass transitions were monitored in separate ion channels: *m/z* 117 > 89 (for HCTL) and *m/z* 136 > 89 (for Hcy) for the thiolactonase assay, and *m/z* 326 > 152 (for VC) and *m/z* 226 > 152 (for acyclovir) for the VC assay. Hcy and HCTL were separated on a Zorbax CN 4.6 × 250mm HPLC column (Dupont Instruments, Cincinnati, OH) using an isocratic mix of 10% methanol and 90% aqueous (0.05% formic acid) with a flow rate of 0.7 mL/min. Acyclovir and VC were separated using the same column, solvents and flow rate, but with an isocratic mix of 20% methanol and 80% aqueous (0.05% formic acid).

## Results

### Purification of human liver HCTLase

**Figure 2:**
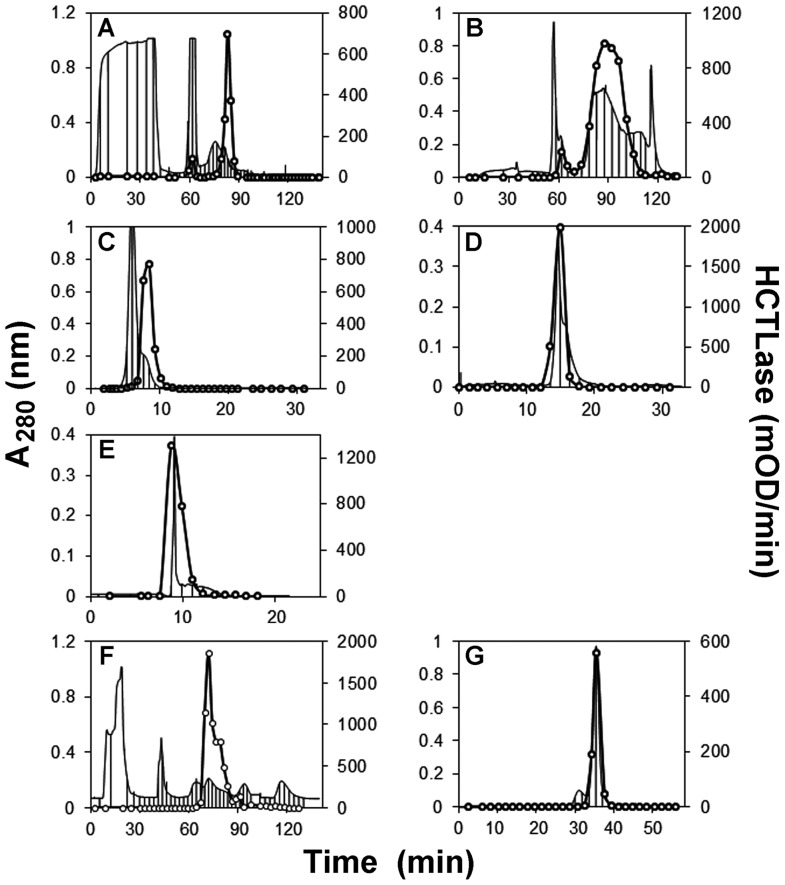
Protein absorbance (A_280_) and HCTLase activity of column fractions from the chromatographic purification of human liver HCTLase (A–E) and recombinant HCTLase (F, G). A_280_ nm, solid lines; HCTL activity, solid lines with open circles. (A) Carboxymethyl (CM) column 1, (B) ceramic hydroxyapatite (HA) column, (C) Superdex 200 column 1, (D) CM column 2, (E) Superdex 200 column 2; (F) HA column, (G) Superdex 200 column. Ticks represent fraction changes.

**Figure 3:**
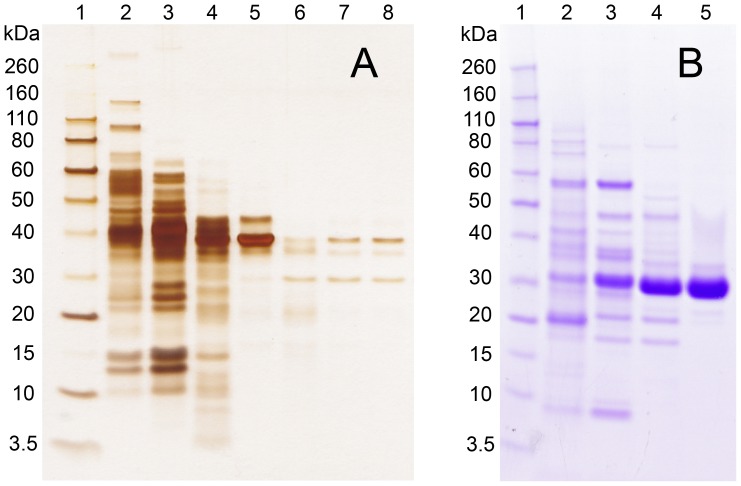
SDS-PAGE analysis of pooled column fractions from (A) human liver and (B) recombinant HCTLase purification. (A) Lane 1, molecular weight markers (kDa); lane 2, liver extract; lane 3, diethylaminoethyl (DEAE) pool; lane 4, CM-1 pool; lane 5, ceramic hydroxyapatite (HA) pool; lane 6, Superdex 200 (#1) pool; lane 7, CM-2 pool; lane 8, Superdex 200 (#2) pool. Gel was stained with silver stain kit (Pierce Chemical). (B) Lane 1, molecular weight markers (kDa); lane 2, *E. coli* extract; lane 3, DEAE pool; lane 4, HA pool; lane 5, Superdex 200 pool. Gel was stained with Imperial Protein Stain (Pierce Chemical).

**Figure 4:**
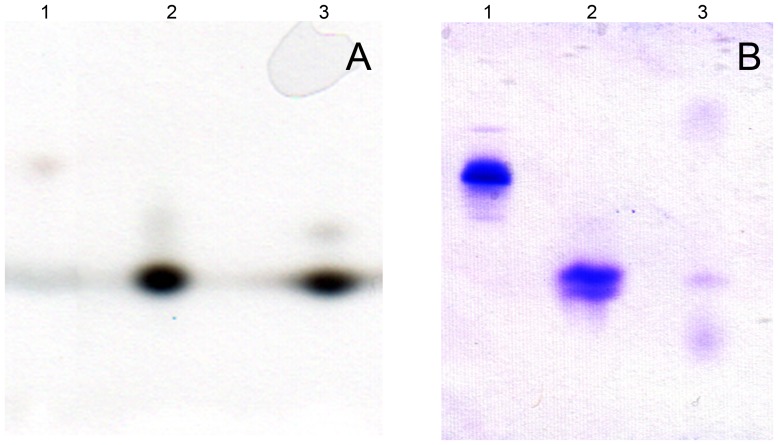
IEF activity and Coomassie stain of liver BPHL and rBPHL. (A) HCTLase activity stain of rBHPL (lane 2) and human liver BPHL (lane 3). Hemoglobin shows a faint band as well (lane 1). (B) Coomassie Blue stain of hemoglobin, rBPHL and human liver BPHL (lanes 1–3, respectively).

Purification steps for human liver HCTLase are shown in Figure 2 A–E (DEAE column trace not shown) and summarized in Table 1. Human liver HCTLase was purified by >100-fold with 5 chromatographic column steps. Three major bands were observed following the final Superdex 200 column. The three bands (Figure 3A, lane 8) were excised for analysis and digested in-gel with trypsin. The extracted peptides were analyzed by liquid chromatography coupled with tandem MS (LC-MS/MS). The peptide mass spectra generated from each band were searched against a human International Protein Index (IPI) peptide database (3/25/09). The higher molecular weight band (≈44 kDa) was identified as acetyl-Coenzyme A acyltransferase 1, with a sequence coverage of 55.2%. The best match for the band at ≈34 kDa was hydroxyacyl-Coenzyme A dehydrogenase, with a 56.4% sequence coverage. The 30 kDa molecular weight protein was identified as BPHL, with sequence coverage of 56.6%. BPHL has a basic isoelectric point of 9.4 [37], consistent with the lack of binding to the anion exchange resin (DEAE Sepharose) and its binding to the cation exchange resin (CM Sepharose). Human liver HCTLase was ≈30 kDa based on elution from the calibrated gel filtration column (Superdex 200). In addition, the activity stain and Coomassie Blue IEF gels confirmed that only the band corresponding to BPHL showed HCTLase activity (Figure 4).

**Table 1:** Purification of human liver HCTLase.

Purification Step	Volume, mL	Total Protein, mg	Total HCTLase activity, Units*	Specific activity, Units/mg	Fold Purification	Yield, %
Liver extract	58.0	435.0	118.3	0.27	–	100
DEAE	58.0	336.4	93.1	0.28	1.0	77.7
CM I	32.0	13.4	73.1	5.50	20.0	62.0
HA	8.0	8.3	48.5	5.80	21.4	41.0
Superdex I	3.5	1.5	41.7	27.90	102.7	34.7
CM II	2.5	0.8	27.6	34.10	125.5	23.4
Superdex II	2.2	0.7	23.6	34.60	127.0	20.0

DEAE indicates diethylaminoethyl; CM, carboxymethyl; and HA, hydroxyapatite.

* Units of HCTLase activity are µmol NTB produced per minute measured at ≈K_m_ concentration of substrate with the partially purified liver BPHL.

### Characterization of purified human liver BPHL

**Figure 5:**
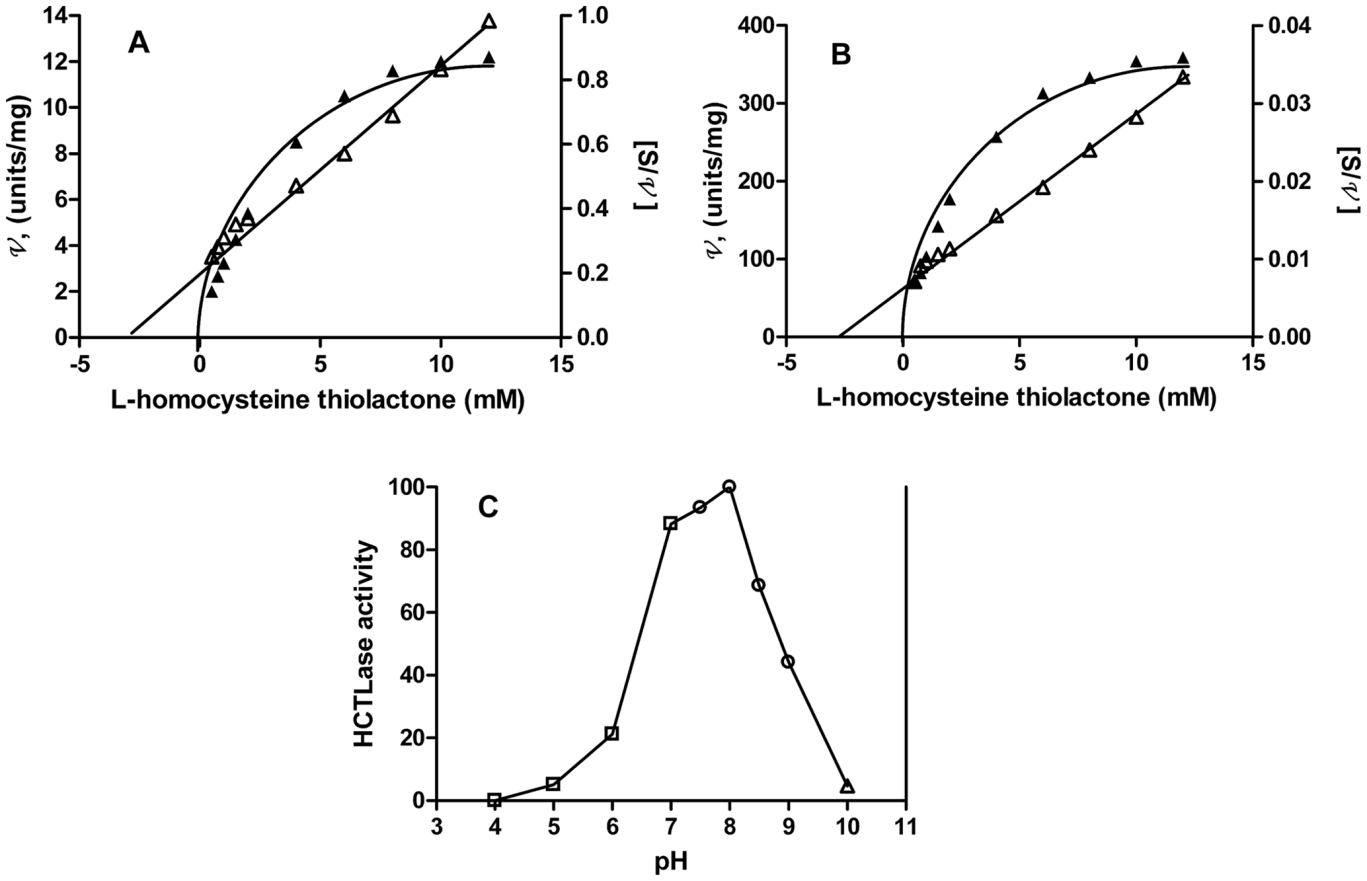
Substrate dependence and pH optimum of HCTL hydrolysis by BPHL. Substrate dependence of HCTL hydrolysis by (A) purified human liver HCTLase (BPHL) and (B) rBPHL. Closed symbols, velocity (v) vs substrate concentration [S]; open symbols, [S]/v vs [S]. K_m_ for HCTL hydrolysis by purified liver BPHL =  3.92 mM and for rBPHL K_m_  =  3.18 mM. (C) HCTLase activity of rBPHL (% maximal activity) as a function of pH. Open squares, □―□ 50 mM citrate/Na_2_HPO_4_; open circles ○―○, 50 mM Tris-HCl; open triangle, ▵―▵ 50 mM Na_2_PO_4_.

Human liver BPHL had a specific activity of 34.6 µmol/min/mg when assayed at 3 mM substrate, a sub-saturating concentration (Table 1). The substrate-dependence of HCTL hydrolysis by the partially purified human liver BPHL revealed a K_m_ value for HCTL hydrolysis of 3.92 mM (Figure 5A). A more detailed characterization of substrate hydrolysis was carried out with the highly purified recombinant BPHL (rBPHL) as described below.

### Inhibition of human liver HCTLase activity

To determine whether liver BPHL is more important in detoxifying HCTL than Blmh or PON1, we incubated human liver homogenates with specific inhibitors for BPHL, Blmh and PON1 and assayed the residual HCTLase activity. The results obtained are summarized in Table 2. The BPHL specific inhibitors valacyclovir and L-proline benzyl ester decreased human liver HCTLase activity by 74% and 88%, respectively. In contrast, Blmh specific inhibitor E-64, a cysteine protease inhibitor, and the two PON1 specific inhibitors EDTA and 2-hydroxyquinoline did not decrease human liver HCTLase activity. Interestingly, iodoacetamide, a reported Blmh inhibitor [8], decreased human liver HCTL hydrolysis by ∼50%. We confirmed that iodoacetamide is a BPHL inhibitor using rBPHL. Following a 10 min incubation of 2 mM iodoacetamide with rBPHL, the HCTLase activity was reduced by 50% (data not shown).

**Table 2:** Inhibition of human liver HCTL activity.

Inhibitor name	% HCTL activity*
No inhibitor	100
*BPHL inhibitors*	
Valacyclovir (20 mM)	25.9
L-Proline benzyl ester (10 mM)	12.0
*Blmh inhibitors*	
E-64 (50 µM)	97.5
Iodoacetamide (2 mM)	57.9 †
*PON1 inhibitors*	
EDTA (10 mM)	141.5
2-hydroxyquinoline (200 µM)	107.0

* HCTLase activity was measured at 10 mM concentration of substrate.

† Iodoacetamide inhibited rBPHL to the same extent.

### Purification of rBPHL

Following identification of BPHL as the most likely candidate responsible for the HCTLase activity, BPHL from a cDNA clone was expressed in *E. coli* and purified as described in the methods section (Figure 2 F-G, DEAE column trace not shown). Highly purified rBPHL, greater than 90% pure as assessed by gel electrophoresis, was obtained after three column chromatographic steps (Table 3). An SDS-PAGE analysis of the purification is shown in Figure 3B. Purified rBPHL had the same molecular weight as human BPHL. To confirm the identity of the purified recombinant protein, it was digested with trypsin and the peptides analyzed by LC-MS/MS. The obtained spectra were searched against the peptide database described above. Human BPHL was the most abundant protein, with a 51.9% sequence coverage.

**Table 3:** Purification of recombinant human BPHL (HCTLase).

Purification Step	Volume, mL	Total Protein, mg	Total HCTLase activity, Units*	Specific activity, Units/mg	Fold Purification	Yield, %
Bacterial extract	18.0	410.0	3802	10.4	–	100
						
DEAE	50.0	64.5	1284	19.9	1.9	34
						
HA	30.0	10.1	911	90.1	8.7	24
						
Superdex	6.5	3.0	857	230.4	22.1	23
						

DEAE indicates diethylaminoethyl; and HA, hydroxyapatite.

* Units of HCTLase activity are µmol NTB produced per minute measured at ≈K_m_ concentration of substrate.

### Characterization of rBPHL activity

**Figure 6:**
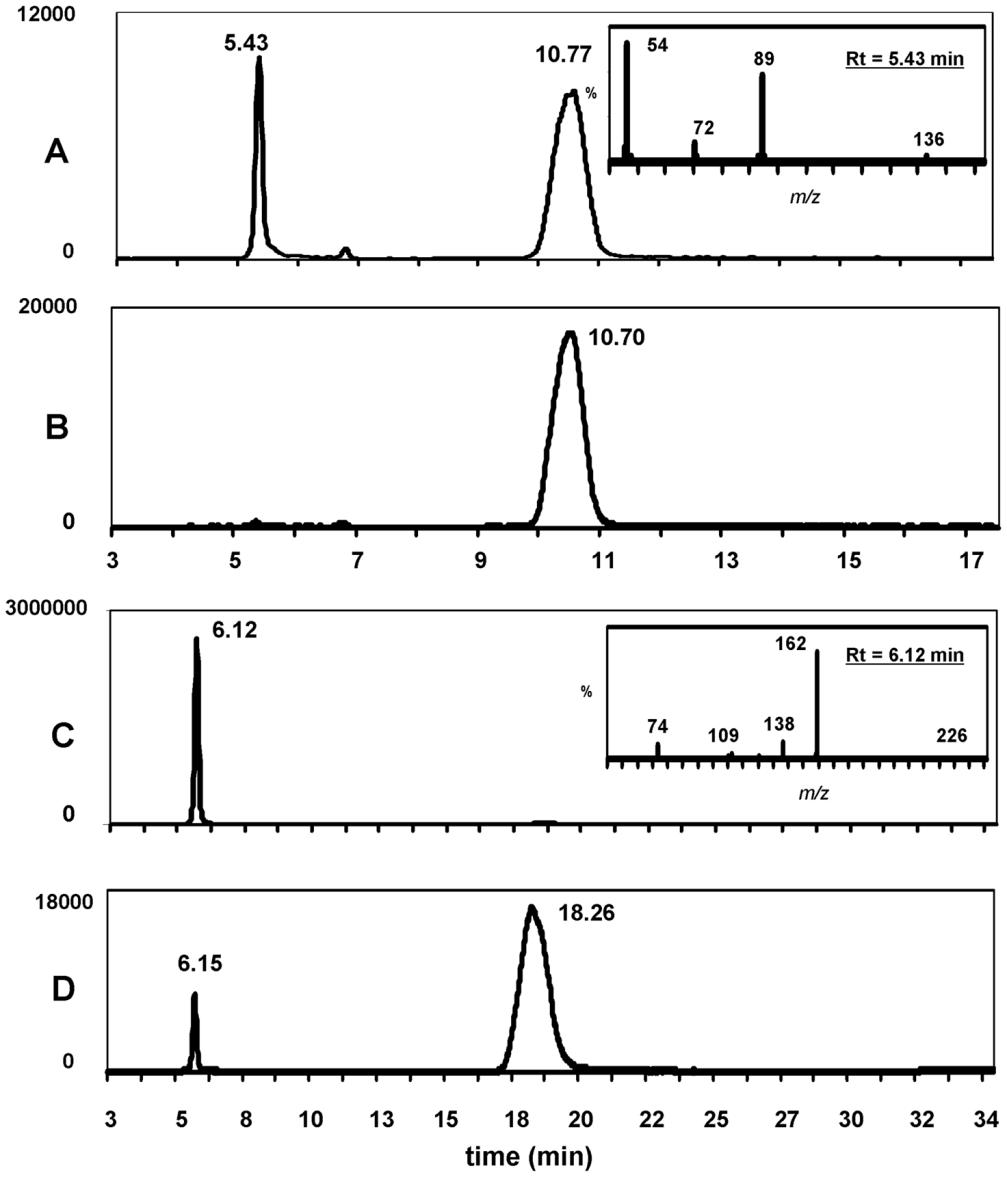
LC-MS chromatograms showing rBPHL-mediated HCTL cleavage at (A) t  =  5 min and (B) t  =  0 of reaction time, and valacyclovir ester cleavage at (C) t  =  5 min and (D) t  =  0. Chromatograms A and B represent the sum of two MRM channels monitoring the following transitions: *m/z* 117 > 89 (for HCTL, Rt  =  10.7 min) and *m/z* 136 > 89 (for Hcy, Rt  =  5.4 min). The inset shows the daughter ion spectrum (for *m/z* 136) of the product peak at Rt  =  5.4 min, and is consistent with the spectrum obtained from commercial Hcy. Chromatograms C and D also represent the sum of two MRM channels monitoring the following transitions: *m/z* 326 > 152 (for VC, Rt  =  18.3 min) and *m/z* 226 > 152 (for acyclovir, Rt  =  6.1 min). The inset shows the daughter ion spectrum (for *m/z* 226) of the product peak at Rt  =  6.1 min, and is consistent with the spectrum obtained from commercial acyclovir. The small product peak at t  =  0 in chromatogram D reflects the extremely rapid metabolism of VC by rBPHL, which generated a detectable amount of acyclovir in even the very short period of time (∼10 sec) between initiation and quenching of the enzymatic reaction.

Purified rBPHL has high HCTLase activity, with a specific activity of 230 µmol/min/mg when assayed at 3 mM substrate (sub-saturating concentration) (Table 3). The K_m_ value for HCTL is 3.18 mM, with a *V*_max_ of 454 µmol/min/mg (Figure 5B), a turnover number of 246.24 s^−1^ (k_cat_) and a catalytic efficiency of 7.7 × 10^4^ M^−1^ s^−1^ (k_cat_/K_m_). The pH optimum of rBPHL was pH 8 (Figure 5C). The ability of rBPHL to hydrolyze HCTL and VC was confirmed by MS (Figure 6 A–D). The BPHL hydrolytic products of HCTL and VC showed the expected reaction products Hcy and acyclovir (Figure 6).

**Figure 7:**
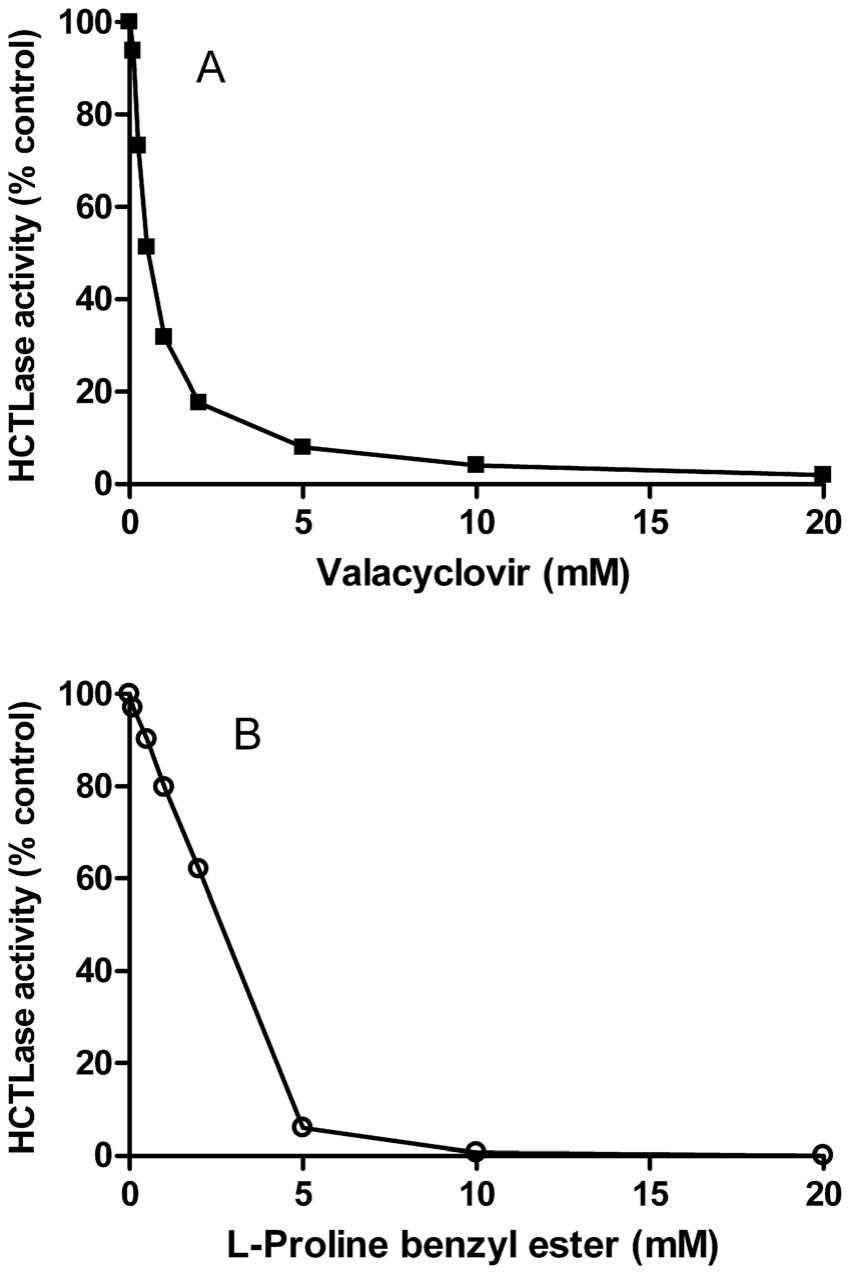
Concentration-dependent inhibition of rBPHL HCTLase by (A) VC and (B) L-proline benzyl ester.

It was surprising to find that GTBL, an alternate substrate used interchangeably for the HCTLase activity of PON1 [21], was not a substrate for BPHL (Table 4). In addition, EDTA, which irreversibly inhibits human PON1, had no effect on BPHL activity at a concentration of 10 mM. Other BPHL substrates, VC and L-proline benzyl ester, the latter reported to be the best substrate for human valacyclovirase [30], inhibited the HCTLase activity of BPHL (Figure 7). Since rBPHL has thiolactonase activity, other PON1 lactone substrates were tested as substrates for rBPHL. Neither clopidogrel or prasugrel thiolactones nor the PON1 lactone substrates dihydrocoumarin and 2-coumaranone were substrates for rBPHL. The substrate specificity for BPHL is shown in Table 4.

**Table 4:** BPHL substrates.

Compound	Specific Activity, Units/mg
L-homocysteine thiolactone	454.0 * †
γ−thiobutyrolactone	<0.001 †
Dihydrocoumarin	<0.001 †
2-coumaranone	<0.001 †
Phenyl acetate	<0.001 †
DL-N-acetyl homocysteine thiolactone	<0.001 †
Clopidogrel thiolactone	<0.02 †
Prasugrel thiolactone	<0.02 †
Valacylovir	103.7 ‡
L-Phe benzyl ester	358.3 ‡
L-Proline benzyl ester	5148.4 ‡
3-Amino-3-phenylpropionic acid ethyl ester	0.6 ‡
L-Ala benzyl ester	1367.7 ‡
β-Ala benzyl ester	1.8 ‡
L-Gly benzyl ester	849.4 ‡
L-Leu benzyl ester	635.0 ‡
L-Val benzyl ester	156.3 ‡
L-Met methyl ester	156.2 ‡
L-Phe benzyl ester	358.3 ‡
L-Tyr benzyl ester	41.4 ‡
L-Trp methyl ester	35.3 ‡
L-Ser methyl ester	484.3 ‡
L-Phe ethyl ester	75.3 ‡

* *V*_max_ from Figure 4B.

† from this study.

‡ from [27].

## Discussion

The link between homocysteinemia and atherosclerosis was established by McCully in 1969 [38]. A number of earlier related studies on hyperhomocysteinemia and hyperhomocysteineuria are cited in the McCully 1969 paper [38] and discussed together with more recent research in excellent reviews [14], [39]. Several biochemical mechanisms appear to contribute to disease risk associated with hyperhomocysteinemia. As pointed out in the recent review by Joseph et al. [14], the relationship of hyperhomocysteinemia and cardiovascular disease is very complex and that the aim of their review was to “…*allow researchers to deconstruct this complex field into separate areas that, when addressed adequately, may lead to findings that elucidate the overall link between hyperhomocysteinemia and cardiovascular disease and allow the design of appropriate clinical trials*…” This report describes the characterization of the highly efficient HCTLase activity of BPHL, a protein previously known only for its role in drug metabolism [28], [29] and provides a more complete understanding of the detoxication pathways for HCTL.

One risk associated with elevated levels of plasma Hcy is related to the formation of disulfide bonds with free cysteine residues on proteins. For example, disulfide bond derivatization of annexin A2 complex by Hcy prevents tissue plasminogen activator binding to A2, resulting in decreased fibrinolytic activity and inhibition of angiogenesis [40]. A second mechanism of protein modification involves the covalent addition of Hcy to the ε-amino groups of lysine residues by HCTL, which can cause protein misfolding, aggregation, loss of function and generation of autoimmune responses [4], [11]. A number of experiments have examined the question of whether Hcy or HCTL is the more toxic species. Studies with yeast strains with mutations in yeast Blmh demonstrated increased sensitivity to HCTL [8] and studies with *Blmh^−/−^* mice also demonstrated increased sensitivity to HCTL [10]. Thus, detoxication of HCTL is an important component in understanding the relationship of hyperhomocysteinemia to vascular and other diseases.

The cardioprotective HCTLase activity had for a number of years been attributed to PON1 [16], then later to Blmh [8]. However, the data reported here strongly suggest that the previously unrecognized HCTLase activity of BPHL is likely to be physiologically more relevant than either PON1 or Blmh for hydrolyzing HCTL and reducing the risk of vascular and other HCTL-associated diseases.

In this regard, it is informative to compare the total HCTL detoxication capacity of plasma PON1 (K_m_, 23 mM) with that of liver HCTLase (K_m_, 3.18 mM). Since many of the HCTLase activities reported for plasma PON1 were carried out with labeled 1 mM HCTL, it makes sense to compare activities at this concentration of substrate. Rates for lower HCTL concentrations are easily extrapolated since 1 mM is below the K_m_ value for both proteins. The following extrapolations are from data reported by Jakubowski [16] and from this study. Assuming an average individual has ≈3 L of plasma with ≈0.0057 µmol HCTL hydrolyzed/min/mL plasma (at 1 mM HCTL) [16] the HCTL detoxication capacity for plasma PON1 of an individual would be ≈17.1 µmol/min/individual. Based on the data from Table 1, 33 g of human liver yielded 118.3 Units of HCTLase (measured at 3 mM HCTL; Units  =  µmol hydrolyzed/min), which would equate to ≈1.195 U/g liver tissue at 1 mM HCTL. Based on an average weight of a human liver of 1,414 g, [41] the total HCTLase Units in a liver would be ≈1,690 µmol/min/liver or 98.8-times the detoxication capacity of plasma PON1. This approximation does not take into account the level of liver PON1 (which in mice represents a small percentage of total PON1) or BPHL levels in kidney and other tissues [26].

It is also useful to compare the catalytic efficiencies of HCTL hydrolysis by Blmh (1 × 10^3^ M^−1^ s^−1^) [8] and PON1 with that of BPHL (7.7 × 10^4^ M^−1^ s^−1^) (this study). Zimny and coworkers reported that the catalytic efficiency of HCTL hydrolysis by Blmh was ≈100-fold higher than that of PON1 [8] indicating that the catalytic efficiency of HCTL hydrolysis by BPHL is almost four orders of magnitude higher than that of PON1 and nearly two orders of magnitude higher than that of Blmh.

Earlier Northern blot expression analyses had shown high expression levels of BPHL in liver and kidney with lower levels in intestine, heart and skeletal muscle [26]. The primary band observed from liver tissue was 1.8 kb with both the 1.8 kb band and a 2.4 kb band visible from kidney tissue. Interestingly, a different expression pattern was observed for Blmh, where low to moderate levels of expression were observed in spleen, prostate, ovary, small intestine, heart, brain, placenta and lung. Elevated levels of expression of Blmh mRNA were observed in testis, skeletal muscle and pancreas with very low expression levels observed in liver, kidney, colon and peripheral blood leukocytes [23]. Thus, tissue expression patterns of BPHL and Blmh are somewhat complementary. In addition, in this study we have shown that the majority of liver HCTLase activity is attributable to BPHL (Table 2).

This study began with the aim of identifying the thiolactonase activity responsible for the second metabolic step in the bioactivation of the platelet inhibitor clopidogrel to the active exo-thiol since it was clear that PON1 was not the physiologically relevant activity for this second step of bioactivation [42], [43]. It has since been shown that both the first and second steps are carried out by cytochrome(s) P450 [43]. PON1 generates an endo-thiol from clopidogrel thiolactone that is not a pharmacologically active metabolite. In the search for a liver thiolactonase that would hydrolyze the intermediate thiolactone metabolite of clopidogrel, we chose HCTL as a convenient thiolactonase substrate. Following purification of the liver HCTLase activity, gel electrophoretic analysis revealed several protein bands of 30–40 kDa (Figure 3A), all too small to be either PON1 or Blmh. MS analysis identified BPHL as the most likely HCTLase candidate. Expression and purification of rBPHL from *E. coli* verified that BPHL did indeed have very high HCTLase activity. BPHL differs from PON1 and Blmh, previously identified HCTLases, in its substrate specificity and more significantly in its high catalytic efficiency. When human liver homogenates were incubated with specific inhibitors for BPHL, Blmh or PON1, HCTLase activity was significantly reduced following incubation with BPHL inhibitors. This finding demonstrates that BPHL is a more relevant liver HCTLase enzyme than the two previously reported Blmh and PON1. The rBPHL did not detectably hydrolyze the thiolactone metabolites of the drugs clopidogrel or prasugrel (another platelet inhibitor) (Table 4).

It is also interesting to compare the substrate specificities of the three identified HCTL hydrolases. Since BPHL metabolized the thiolactone HCTL, we tested it with several other PON1 substrates. The rBPHL failed to hydrolyze dihydrocoumarin, 2-coumaranone, phenyl acetate and DL-*N*-acetyl homocysteine thiolactone (Table 4). GTBL is often used as a surrogate for measuring HCTL hydrolysis; however, BPHL does not hydrolyze GTBL. Many investigators cite problems of albumin interference with the Ellman assay and degradation of HCTL at alkaline pH as a rationale for using GTBL, which has been used as a lactonase substrate for analysis of PON1 activity [3], [5], [21], [22], [44]. GTBL is used in a commercially-available assay expressly for measuring serum HCTLase, using Ellman's reagent. However, BPHL has no GTBLase activity. Blmh, an aminopeptidase, also catalyzes the hydrolysis of HCTL but not the hydrolysis of GTBL. In previous studies, typical esterase (*p*-nitrophenyl acetate, *p*-nitrophenyl butyrate) and peptidase substrates (amides such as Lys-*p*-nitroanilide) were tested as BPHL substrates [29]. BPHL had almost no activity for any of these substrates except for *p*-nitrophenyl butyrate, for which it had only low activity. The results from this study show BPHL to be an efficient HCTLase but not a general lactonase. Studies by Kim et al [28], [29], provide additional information on the substrate specificity of BPHL. It is worth noting the structural similarities between HCTL and VC as both possess a free amino group on the carbon alpha to the carbonyl that is neutralized by the enzyme's active site residue D123. Lai et al. described BPHL as a specific alpha-amino acid ester hydrolase [30].

Prior to the studies reported here, a physiological activity of BPHL was unknown [30], [31], but due to its localization to the liver and kidney using Northern blots, BPHL was hypothesized to be involved in detoxification and drug metabolism [26]. The discovery of BPHL's role in converting the prodrug VC to acyclovir identified one such pathway [28]. It is interesting that both Blmh [23] and BPHL [28] were first characterized as drug metabolizing enzymes. The role of PON1 in the metabolism of some drugs has also been characterized (reviewed in [45]). Since proteins did not evolve to metabolize drugs, the high HCTLase activity of BPHL reported here provides evidence that BPHL is most likely a primary detoxication pathway for HCTL and its high degree of sequence conservation across species would indicate that this HCTLase activity of BPHL is a universally shared function.
